# Transcriptomic Analysis Reveals New Insights into High-Temperature-Dependent Glume-Unclosing in an Elite Rice Male Sterile Line

**DOI:** 10.3389/fpls.2017.00112

**Published:** 2017-02-14

**Authors:** Chongyun Fu, Feng Wang, Wuge Liu, Dilin Liu, Jinhua Li, Manshan Zhu, Yilong Liao, Zhenrong Liu, Huijun Huang, Xueqin Zeng, Xiaozhi Ma

**Affiliations:** ^1^Rice Research Institute, Guangdong Academy of Agricultural SciencesGuangzhou, China; ^2^Guangdong Provincial Key Laboratory of New Technology in Rice BreedingGuangzhou, China

**Keywords:** rice, glume-unclosing, post-anthesis, high temperature, transcriptome

## Abstract

Glume-unclosing after anthesis is a widespread phenomenon in hybrid rice and also a maternal hereditary trait. The character of Glume-unclosing in rice male sterile lines also seriously influences germination rate and the commercial quality of hybrid rice seeds. We validated that the type of glume-unclosing after anthesis in the elite rice thermo-sensitive genic male sterile (TGMS) line RGD-7S was caused by high temperature. Transcriptomic sequencing of rice panicles was performed to explore the change of transcript profiles under four conditions: pre- and post-anthesis under high temperature (HRGD0 and HRGD1), and pre- and post-anthesis under low temperature (LRGD0 and LRGD1). We identified a total of 14,540 differentially expressed genes (DEGs) including some heat shock factors (HSFs) across the four samples. We found that more genes were up-regulated than down-regulated in the sample pair HRGD1vsHRGD0. These up-regulated genes were significantly enriched in the three biological processes of carbohydrate metabolism, response to water and cell wall macromolecular metabolism. Simultaneously, we also found that the HSF gene *OsHsfB1* was specially up-regulated in HRGD1vsHRGD0. However, the down-regulated DEGs in LRGD1vsLRGD0 were remarkably clustered in the biological process of carbohydrate metabolism. This suggests that carbohydrate metabolism may play a key role in regulation of glume-unclosing under high temperature in RGD-7S. We also analyzed the expression pattern of genes enriched in carbohydrate metabolism and several HSF genes under different conditions and provide new insights into the cause of rice glume-unclosing.

## Introduction

Rice (*Oryza sativa*) is one of the main stable food crops in the world. The successful development of rice cytoplasmic male sterile (CMS) line made the hybrid rice seed production possible in the 1970s. Cheng et al. (2007) reported a 10–20% increase in yield by the introduction of hybrid rice compared to conventional varieties. The regulation of rice floret opening and closing is a very important characteristic for hybrid rice seed production. However, in recent years, glume-unclosing after anthesis is found to be a widespread phenomenon in the production of hybrid rice seeds and also an inherited characteristic of rice sterile lines (Wang et al., 1991). Several rice male sterile lines widely used including thermo-sensitive genic male sterile (TGMS) lines, such as RGD-7S, Long S and 85S, and CMS lines, such as Fengtian A, Guang 8A, Fuyi A, and Sanxiang A (Liu et al., 2008), showed serious glume-gapping in the seed production of hybrid rice in China. Glume-unclosing not only reduces germination rate, quality and storage life of hybrid rice seeds, but also influences the commercial value of rice hybrid seeds.

The opening and closure of rice glumes is controlled by swelling and subsequent withering of lodicules. The swelling of rice lodicules resulted from water uptake and cell wall loosening of lodicules causing cell expansion (Wang and Gu, 2012). Cell expansion is the predominant process for flower opening and is also a combined process of cell wall weakening, carbohydrate allocation and water uptake (Christiaens et al., 2016). Several factors, such as carbon dioxide, temperature, humidity, metal ions, and plant hormones, have been reported to influence the opening and closure of florets (Kalika et al., 2005; Yuan et al., 2009). The significant accumulation and outflow of soluble sugars and ions are found in the lodicules in the course of the floret opening and closure (Wang et al., 1991, 1994; Heslop-Harrison and Heslop-Harrison, 1996). Plant hormones, including jasmonate (JA), methyl jasmonate (MeJA), and ethylene, have been shown to affect the opening of rice florets (He et al., 2012). Cytological analysis found that smaller vascular bundles were observed in the lodicules of the glume-opening mutants or glume-gapping CMS lines compared with those of the wild type or the relative maintainer lines (Liu et al., 2008; Liao et al., 2015).

However, the knowledge of rice glume opening and closure, especially the dependence on temperature, still remains limited. In rice, three opening-glume mutants *osjar1, osjar2* (Riemann et al., 2008; Xiao et al., 2014), and *ucgl* (Liao et al., 2015) were identified to be not influenced by high temperature. Their phenotypes of open-staying florets are associated with jasmonic acid biosynthesis. On the contrary, the cleistogamy or chasmogamy of the cleistogamous rice mutant *spw1-cls* is dependent on temperature (Koike et al., 2015). In addition, in barley, the *cleistogamy 1*(*Cly 1*) gene, encoding a transcription factor containing two AP2 domains, regulates the closed floret habit. *Cly1* contains a putative microRNA (miR172) target site, which suggests that it is potentially regulated by miR172. Sequence variations at three base positions within the miR172 target sequence are found in 274 barley varieties (Nair et al., 2010). In noncleistogamous varieties, the C*yl1* mRNA is degraded by miR172 to allow the lodicules to swell; in contrast, the *Cly1* mRNA is not cleaved by miR172 causing the failure of the lodicules to expand in cleistogamous varieties (Wang et al., 2015).

Although it is known that temperature influences the opening and closure of rice glumes, it remains unclear how temperature affects the closure of rice glumes after anthesis. We conducted transcriptomic sequencing of rice panicles at two stages (pre- and post-anthesis) under high and low temperatures with following objectives: (i) to understand the major biological processes involved in regulation of glume-unclosing after anthesis; (ii) to characterize the expression pattern of genes associated with glume-unclosing under high temperature. Our results provide valuable clues for hybrid rice breeding and genetic improvement.

## Materials and methods

### Plant materials and phenotype investigation

RGD-7S, developed by pyramiding the blast resistant genes *Pi1, Pi2*, and *Pita* through marker-assisted selection, is an elite *indica* rice thermo-sensitive genic male-sterile line that has been widely used in hybrid rice production in China. RGD-7S carries strong rice blast resistance, good grain quality, and high combining ability.

RGD-7S was grown in the Dafeng Experimental Station, Guangzhou in July, 2015. Pollens of RGD-7S are fertile when grown at lower than 21°C. Hence, to rule out the interference from the fertility change, the low temperature limit was set at 21°C in this study. Four plants at 60 days old were transplanted in each of two plastic casks. After recovery for 1 week, one cask was transferred to 33.5°C (39°C day and 28°C night for 12 h) and another to 24.5°C (28°C day and 21°C night for 12 h), and stayed for 4 weeks in the two separate chambers with different temperatures (relative humidity: 90%) (Sanyo, MLR-351H). One week after anthesis, the percentage of glume-opening was scored using 10 panicles under each temperature condition.

### Sample preparation and sequence library construction

Panicles were harvested 2 days before anthesis and 1 week after anthesis, respectively. The samples were named as HRGD0 (pre-anthesis under high temperature), HRGD1 (post-anthesis under high temperature), LRGD0 (pre-anthesis under low temperature), and LRGD1 (post-anthesis under low temperature), respectively. About three biological replicate samples (1.5 g each), each containing three panicles from different plants, were collected. All of the collected panicles were stored in liquid nitrogen until RNA isolation. Total RNA samples were extracted using the TRIzol reagent (Invitrogen) and then treated by RNase-free DNase I (Takara) to remove genomic DNA. mRNA libraries were constructed according to the standard protocols provided by Illumina. The quality of mRNA including purity, quantity and integrity was tested using Nanodrop, Qubit, and Agilent 2100. mRNA was enriched using Dynabeads oligo (dT) (Dynal; Invitrogen) and fragmented using fragmentation buffer. Double-stranded cDNAs were synthesized using reverse transcriptase (Superscript II; Invitrogen) and random hexamer primers and further purified using AMPure XP beads. Finally, the purified double-stranded cDNA samples were further enriched by PCR to construct the final cDNA libraries that were sequenced using Hiseq 2500 (150 bp paired ends) by Novogene (China). All raw-sequence reads data were uploaded in NCBI Sequence Read Archive (SRA, http://www.ncbi.nlm.nih.gov/Traces/sra) with accession number SRA414710.

### Mapping sequencing results and differential expression analysis

Adaptor sequences and low-quality sequences were removed from the raw reads (*Q* < 20). Clean reads were aligned to reference genome sequences of the *Japonica* rice Nipponbare genome (http://ftp.ensemblgenomes.org/pub/release-24/plants/fasta/oryza_sativa/dna/Oryza_sativa.IRGSP-1.0.24.dna.toplevel.fa.gz) using TopHat (v2.0.12). The default parameters were used, allowing mismatches of no more than two bases. Reference-based assembly of all the reads was performed using the Cufflinks v2.1.1 reference annotation based transcript (RABT) assembly method. The assembled transcript fragments were compared with the reference annotation to predict new genes, novel exons and optimization of gene structures using Cuffcompare.

Gene expression differences in the different sample pairs were detected using the DESeq package (v1.10.1). In this study, to investigate the genes involved in the opening and closure of rice florets, the four sample pairs HRGD1vsHRGD0, HRGD1vsLRGD1, HRGD0vsLRGD0, and LRGD1vsLRGD0 were used to investigate the differentially expressed genes under different conditions, including between pre- and post-anthesis under high temperature, post-anthesis between high and low temperatures, pre-anthesis between high and low temperatures, and between pre- and post-anthesis under low temperature. The *P*-value threshold was determined using the false discovery rate (FDR) in multiple tests (Benjamin and Hochberg, 1995). The thresholds were set using an FDR ≤ 0.05 and the absolute value of log2 (Fold change) with FPKM ≥ 1 to determine significant differences in gene expression. The FPKM (reads per kb per million reads) was used to eliminate the influence of different gene lengths and sequencing discrepancies on the quantification of gene expression to ensure direct comparison of gene expression in different sample pairs (Mortazavi et al., 2008).

### Functional classification of differentially expressed genes

Analysis of functional enrichment including Gene Ontology (GO) was performed to identify which DEGs were significantly enriched in GO terms. GO enrichment of DEGs was conducted using the GOseq R package (Release2.12). GO terms with corrected *P* < 0.05 were considered remarkably enriched by differentially expressed genes. The GO annotations were functionally classified using the WEGO software for gene function distributions. KOBAS software (v2.0) was used to identify the statistical enrichment of differentially expressed genes in KEGG pathways. The pathways with an FDR value of less than 0.05 were regarded as those with genes showing significantly differential expression.

### Real-time PCR confirmation of differentially expressed genes

A total of 31 differentialy expressed genes were randomly selected to confirm the expression level of RNA-seq results using quantitative reverse transcription PCR (RT-qPCR). The corresponding sequences of these genes were obtained from the rice genome sequence database (Rap-db). The RT-qPCR primers were designed according to the transcript sequences of these genes using Primer3 software (http://frodo.wi.mit.edu/) (Table S1). *Osactin1* gene was used as the internal control. The three biological replicate samples same to the samples for RNA-seq were equally mixed to extract RNA. First-strand cDNA was synthesized from 1 mg of DNase I-treated RNA samples in a 20-μl reaction solution with random primers, using a ReverTra Ace-akit (TOYOBO). Standard RT–qPCR was performed using SYBR Green SuperMix (Bio-Rad) on a CFX96 Real Time System (BioRad).

## Results

### High temperature causes rice glume-unclosing

**Figure 1:**
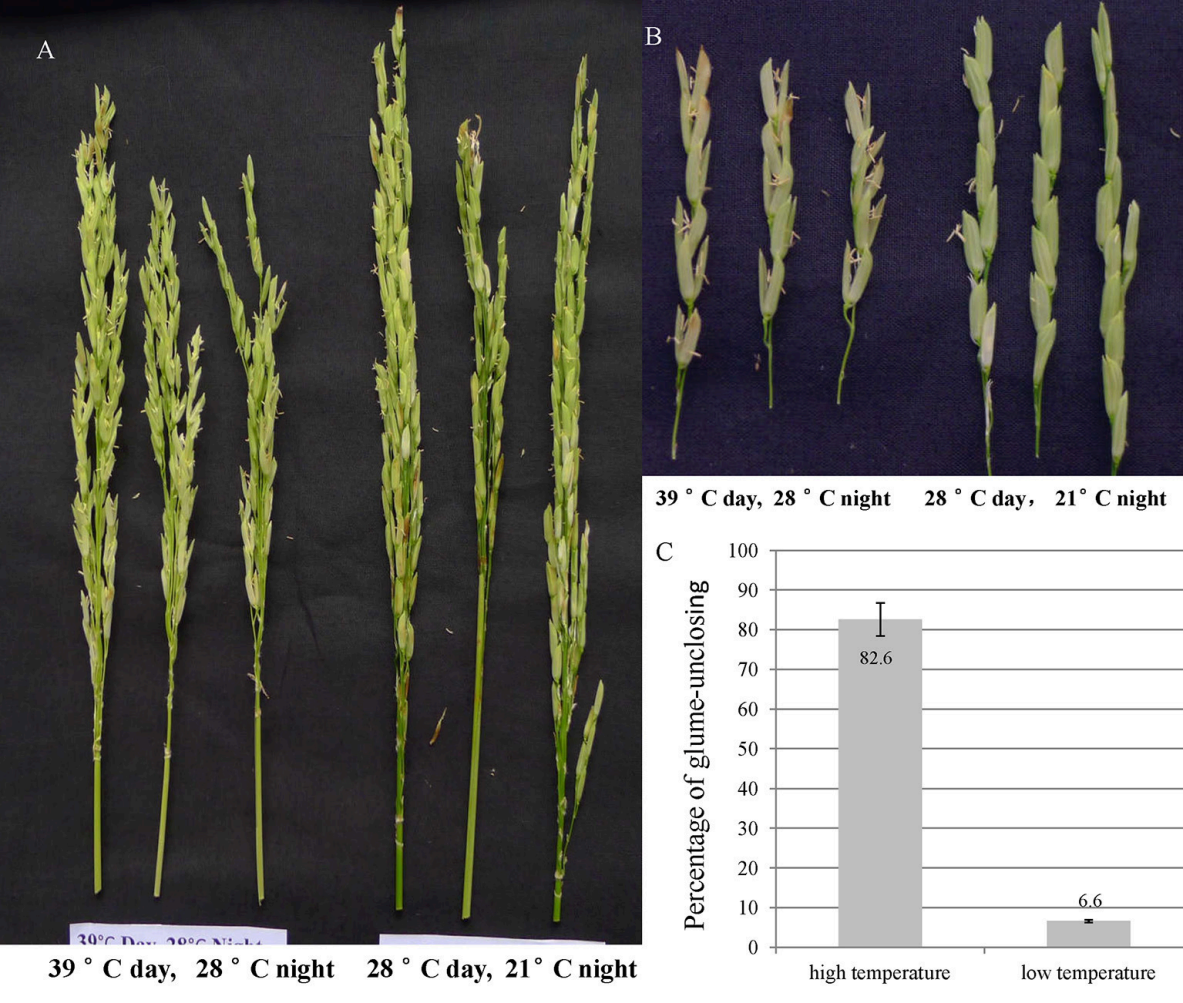
**The rice glume-unclosing phenotype 1 week after anathesis under different temperatures and its statistics**. **(A,B)** the phenotype of rice glume-unclosing after anthesis under different temperature. **(C)** The statistics of rice glume-unclosing under different temperature.

In order to explore the temperature effect on glume-closing, the florets from 10 random flowered panicles were counted for their glume-unclosing rate under the two different temperature conditions. The percentage of glume-unclosing was 82.6% under high temperature and was 6.6% under low temperature (Figures 1A–C). Under low temperature, most of unclosed glumes occurred only on the top of rice panicles. These results indicate that high temperature significantly influences the closure of rice glumes after anthesis.

### Transcriptomic profiles of rice panicles

**Figure 2:**
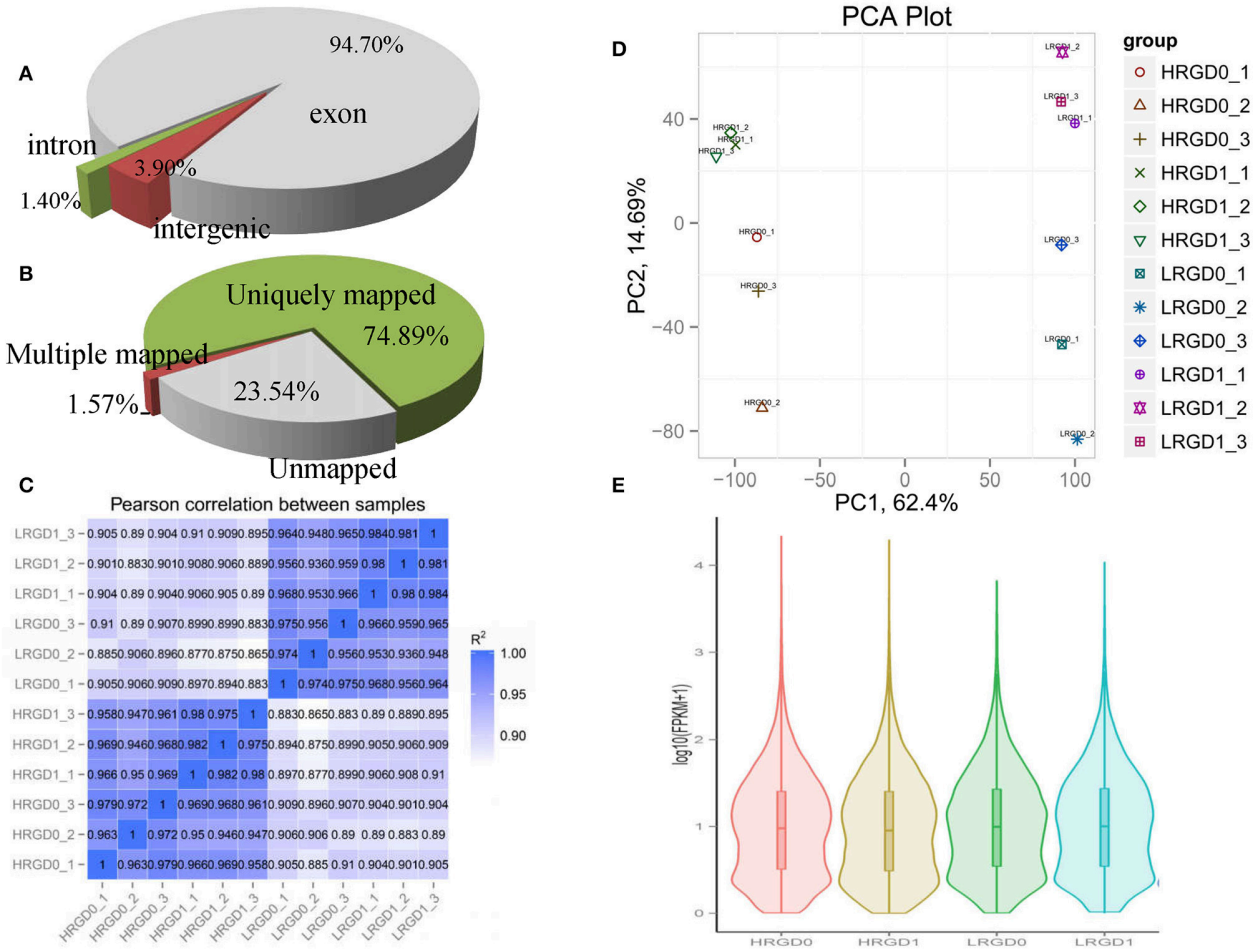
**Distribution and quality of reads in different samples**. **(A)** The distribution of overall mapped reads for all samples in the different regions. **(B)** Overall results of paired-end reads (PEs) for all samples mapped to the reference genome. **(C)** The correlation analysis between samples. **(D)** The principal component analysis (PCA) among biological replicates. **(E)** The gene expression distribution (FPKM) in four samples.

To comprehensively assess the change of transcript profile influenced by temperature, pre- and post-anthesis panicles under different temperatures were harvested for RNA-Seq. The numbers of raw reads of the 12 samples range from 44 to 59 million with an error rate of approximately 0.02%, yielding 6.16–8.25G clean bases (Table S2). The percentages of mapped reads are 94.7, 1.4, and 3.9% in exon, intron, and intergenic region, respectively (Figure 2A). About 76.5% total reads are mapped to the reference genome. Among them, uniquely mapped reads are about 74.89% of total reads and multiple mapped reads are 1.57% of them (Figure 2B, Table S3). The correlation analysis between samples and the principal component analysis (PCA) among biological replicates were performed to assess the overall quality of RNA-seq data. All of the correlation coefficients between biological replicate samples are greater than 0.95 (Figure 2C, Table S4) and the three biological replicates, especially at the post-anthesis, were significantly clustered (Figure 2D), indicating that expression patterns between samples have high similarity and the sequencing data may be used to analyze differential expression of genes. The expression levels of genes were estimated using fragments per kilobase of transcript per million mapped fragments (FPKM) (Trapnell et al., 2010). The expression levels of genes showed similar tendency among the four samples, and most genes had low expression levels (Figure 2E). The RT-qPCR results were in good agreement with the RNA-seq results (Table 1).

**Table 1:** **The expression value of 31 selected genes in RNAseq and real time PCR**.

**Gene ID**	**Gene description**	**RNAseq (FPKM)**				**Real time PCR (FC)**			
		**LRGD0**	**LRGD1**	**HRGD0**	**HRGD1**	**LRGD0**	**LRGD1**	**HRGD0**	**HRGD1**
OS11G0700900	Glycoside hydrolase	1.27	7.3	14.04	84.38	1	3.54	0.14	2.98
OS09G0389000	Conserved hypothetical protein	0.06	0.07	0.21	0.9	1	1.12	4.9	11.09
OS11G0702100	Similar to Class III chitinase homolog	1.08	8.95	1.16	27.19	1	1.84	0.18	5.86
OS12G0151500	Similar to Alpha-2,8-sialyltransferase 8B	17.68	12.27	13.82	4.86	1	1.14	1.71	3.67
OS03G0642300	Protein kinase-like domain containing protein	1.76	1.23	8.37	2.41	1	0.44	1.9	0.91
OS05G0332300	Similar to CBL-interacting protein kinase 2	18.29	16.41	7.15	2.38	1	0.33	0.84	0.36
OS02G0733300	Similar to Endo-beta-1,4-glucanase precursor	13.56	1.03	1.91	0.23	1	0.01	0.18	0
OS03G0277300	Heat shock protein 70	6.39	7.27	23.6	42	1	1.06	4.25	8.9
OS01G0840100	Heat shock protein Hsp70 family protein	795.62	346.5	51.29	93.25	1	0.66	1.43	1.74
OS01G0746700	Similar to Mannan endo-1,4-beta-mannosidase 2	87.57	23.52	27.62	10.16	1	0.15	0.71	0.05
OS11G0701800	Chitinase	0.69	1.13	1.76	11.52	1	3.43	7.92	36.7
OS12G0554800	Similar to Polygalacturonase-like protein	5.28	5.59	4.63	2.07	1	0.71	1.02	0.56
OS01G0713200	Similar to Beta-glucanase	51.96	59.84	11.57	95.37	1	1.56	1.23	7.02
OS11G0701000	Class III chitinase homolog	0.04	0.5	0.44	1.84	1	3.2	0.19	3.12
OS03G0828300	Similar to (1-4)-beta-mannan endohydrolase-like protein	5.28	6.64	3.08	18.04	1	2.47	0.7	1.39
OS08G0518900	Chitinase	0.2	2.21	0.39	3.08	1	3.87	1.15	7.73
OS07G0106200	Similar to Hexose transporter	26.09	71.61	41.67	187.08	1	2.06	0.22	3.3
OS09G0538700	Protein of DUF632 domain containing protein	5.34	4.2	3.64	1.74	1	0.33	0.63	0.33
OS01G0860450	Hypothetical protein	0.5	0.05	2.68	0.82	1	0.31	2.67	2.65
OS05G0460000	Similar to 70 kDa heat shock cognate protein 1	262.72	167.24	47.67	101.44	1	0.59	1.55	1.49
OS03G0276500	Similar to Heat shock protein 70	259.79	217.23	24.51	42.77	1	0.57	0.79	0.47
OS08G0244500	Similar to hydrolase	1.41	4.04	3.1	8.77	1	1.52	1.11	1.16
OS11G0701200	Glycoside hydrolase, family 18 protein	0.49	2.61	0.28	5.76	1	1.02	0.51	3.17
OS11G0701400	Chitinase	0.41	1.38	0.08	4.33	1	1.04	1.08	1.12
OS11G0702200	Glycoside hydrolase	0	0.25	0.02	1.15	1	0.71	7.31	18.22
OS04G0376400	Glycoside hydrolase, family 18	1.23	1.03	1.1	0.24	1	0.64	1.51	2.04
OS08G0445700	HAD-superfamily hydrolase subfamily IIB protein	9.01	12.68	17.91	69.71	1	1.34	0.6	1.23
OS04G0513400	Similar to Beta-glucosidase	15.46	74.75	0.73	3.66	1	4.13	0.33	1.56
OS11G0701100	Similar to Class III chitinase homolog	0.06	0.49	0.05	3.83	1	1.37	8.86	30.46
OS04G0486950	Similar to Malate synthase	5.2	11.65	12	45.73	1	10.75	17.52	27.03
OS05G0247100	Similar to Glycosyl hydrolases family 18	758.77	811.8	468.75	1363.79	1	1.48	0.76	1.78

### Analysis of differentially expressed genes (DEGs) under different temperatures

**Figure 3:**
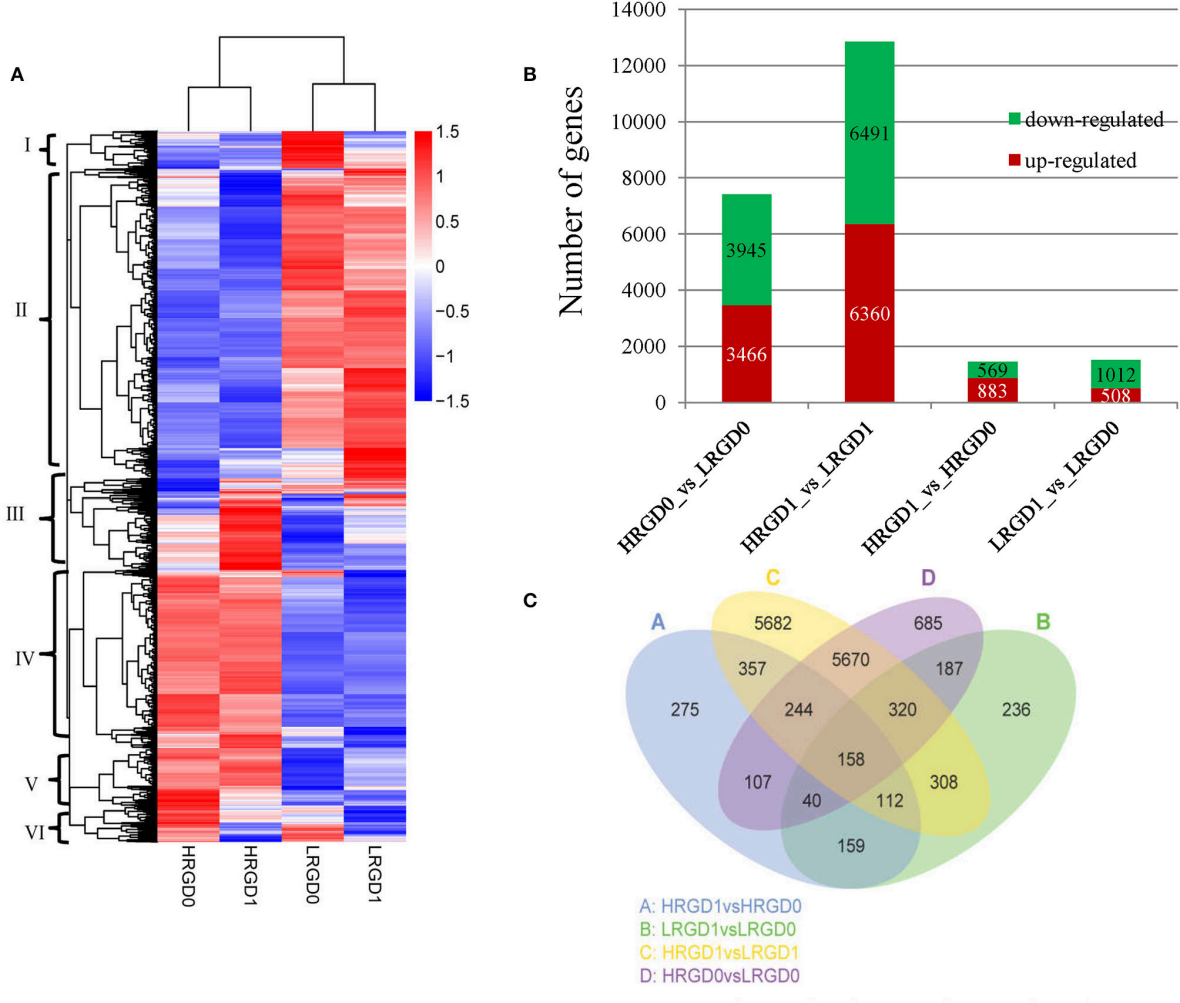
**The DEGs in four samples**. **(A)** Heat map of scaled FPKM values in four samples HRGD0, HRGD1, LRGD0, and LRGD1. Red: high expression; Blue: low temperature. **(B)** The number of up- and down-regulated DEGs in four sample pairs HRGD1vsHRGD0, LRGD1vsLRGD0, HRGD1vsLRGD1, and HRGD0vsLRGD0. **(C)** Venn diagram of the numbers of expressed genes in sample pairs HRGD1vsHRGD0, LRGD1vsLRGD0, HRGD1vsLRGD1, and HRGD0vsLRGD0.

In order to understand the genes regulated by temperature at the stages of rice floral development (pre- and post-anthesis), we performed comparative analysis of differentially expressed genes (DEGs) in the four sample pairs (HRGD1vsLRGD1, HRGD1vsHRGD0, LRGD1vsLRGD0, and HRGD0vsLRGD0). A total of 14,540 DEGs were divided into 6 groups (Figure 3A). We found that high temperature greatly influenced differential patterns of gene expression at the stages of both pre- and post-anthesis, and detected 12,851 DEGs (6491 down-regulated and 6360 up-regulated) in HRGD1vsLRGD1 and 7411 DEGs (3945 down-regulated and 3467 up-regulated) in HRGD0vsLRGD0, respectively (Figure 3B). The numbers of DEGs were similar under high and low temperatures. Interestingly, we detected more up-regulated genes than down-regulated genes only in HRGD1vsHRGD0, indicating that some genes may be specifically up-regulated under high temperature at the stage of post-anthesis.

To characterize the expression pattern of genes regulated by high temperature at the stages of pre- and post-anthesis, we analyzed the overlapped DEGs in different combination of sample pairs and identified 158 DEGs shared in the four samples (Figure 3C). About 705 DEGs overlapped in HRGD0vsLRGD0 and LRGD1vsLRGD0 that may be specifically regulated by high temperature at the stage of pre-anthesis. Similarly, 871 DEGs were shared in HRGD1vsLRGD1 and HRGD1vsHRGD0 that may be associated with glume-unclosing after anthesis under high temperature.

**Figure 4:**
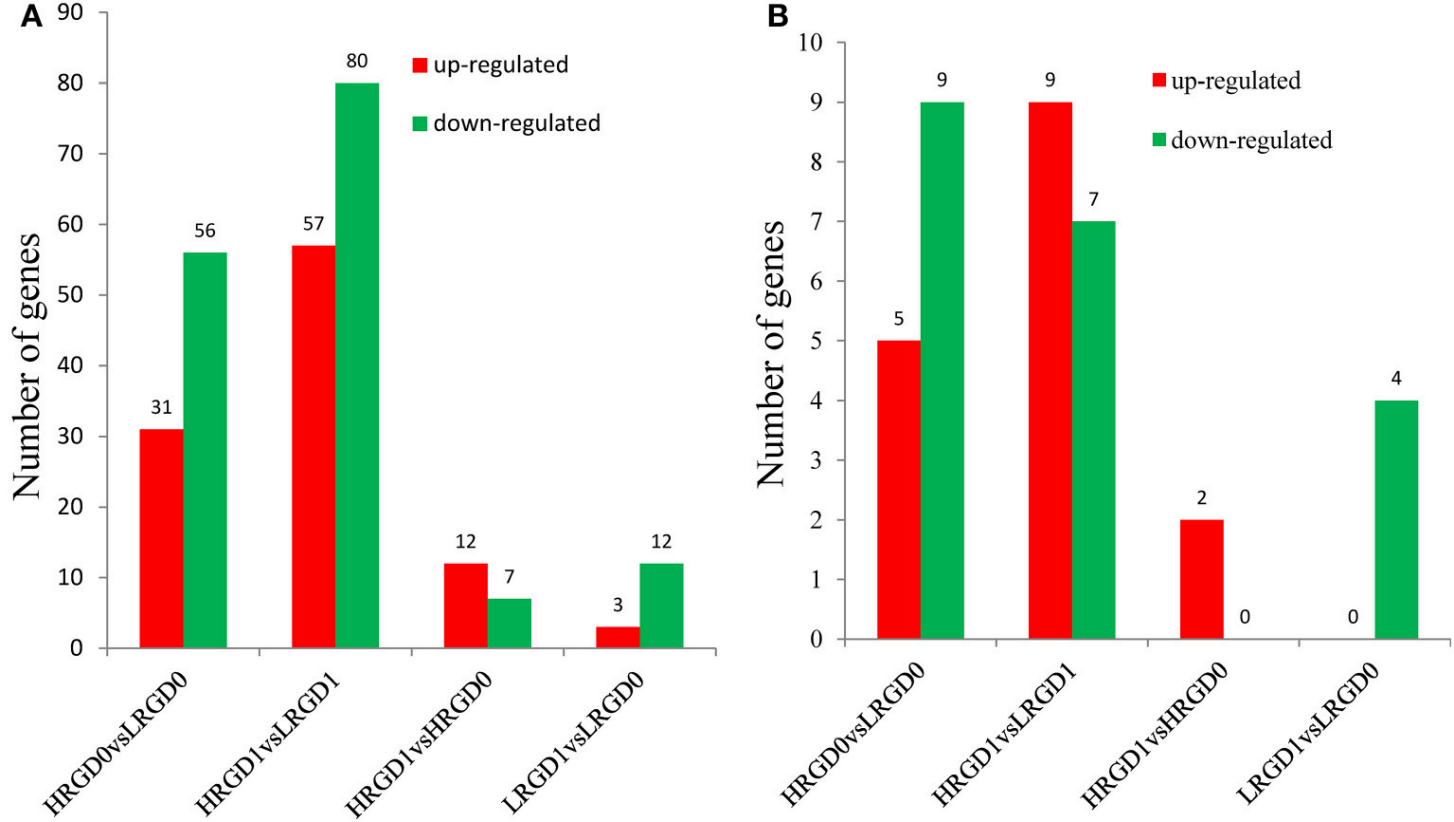
**The differentially expressed transcription factors (TFs). (A)** The number of up- and down regulated TFs in four sample pairs. **(B)** The number of up-and down-regulated heat shock factors (HSFs) in four sample pairs.

Transcription factors (TFs) activate or repress the transcription of downstream target genes by directly binding to the promoters of target genes in a sequence-specific manner (Qu and Zhu, 2006). Plants respond to heat stress by enhancing the expression of genes encoding heat shock proteins (HSPs) through activation of heat shock factors (HSFs) recognizing the heat shock elements (HSEs) in the promoter region of HSPs (Chauha et al., 2011). Transcription factors, especially HSFs were investigated in these DEGs. We detected 87 TFs (31 up-regulated and 56 down-regulated) in HRGD0vsLRGD0, 137 TFs (57 up-regulated and 80 down-regulated) in HRGD1vsLRGD1, 19 TFs (12 up-regulated and 7 down-regulated) in HRGD1vsHRGD0, and 15 TFs (3 up-regulated and 12 down-regulated) in LRGD1vsLRGD0 (Figure 4A). In the four sample pairs, we found that the up-regulated TFs were more than the down-regulated TFs only in HRGD1vsHRGD0. We further analyzed HSFs in these TFs, and detected 14 HSFs (5 up-regulated and 9 down-regulated) in HRGD0vsLRGD0, 16 HSFs (9 up-regulated and 7 down-regulated) in HRGD1vsLRGD1, 2 up-regulated TSFs in HRGD1vsHRGD0 and 4 down-regulated TSFs in LRGD1vsLRGD0 (Figure 4B, Table 2). In HRGD1vsLRGD1 and HRGD1vsHRGD0, the up-regulated HSFs were more than down-regulated HSFs, suggesting that some HSFs should be specifically up-regulated at the stage of post-anthesis under high temperature.

**Table 2:** **The differentially expressed heat shock factors in four sample pairs**.

**Sample pair**	**Gene ID**	**Change**	**Gene description**
HRGD0vsLRGD0	OS01G0733200	up	Similar to Heat shock transcription factor 29
	OS02G0527300	up	Similar to Heat shock transcription factor 31
	OS03G0366800	up	Similar to Heat stress transcription factor B-4d
	OS06G0565200	up	Putative heat stress transcription factor A-6a
	OS09G0526600	up	Similar to Isoform 2 of Heat stress transcription factor B-2c
	OS02G0232000	down	Similar to Heat shock transcription factor 29
	OS03G0745000	down	Similar to Heat stress transcription factor A-2a
	OS04G0568700	down	Similar to Heat stress transcription factor Spl7
	OS05G0530400	down	Heat stress transcription factor Spl7
	OS06G0553100	down	Similar to Heat stress transcription factor C-2b
	OS07G0178600	down	Similar to Heat shock transcription factor 29
	OS07G0640900	down	Similar to Isoform 2 of Heat stress transcription factor B-4b
	OS09G0455200	down	Heat shock factor (HSF)-type, DNA-binding domain containing protein
	OS09G0456800	down	Similar to Heat stress transcription factor Spl7
HRGD1vsLRGD1	OS01G0733200	up	Similar to Heat shock transcription factor 29
	OS02G0527300	up	Similar to Heat shock transcription factor 31
	OS03G0224700	up	Similar to HSP protein
	OS03G0366800	up	Similar to Heat stress transcription factor B-4d
	OS03G0795900	up	Heat stress transcriptioon factor, High-temperature stress tolerance, Tolerance to environmental stresses
	OS06G0565200	up	Putative heat stress transcription factor A-6a
	OS08G0546800	up	Similar to Heat stress transcription factor B-2b
	OS09G0526600	up	Similar to Isoform 2 of Heat stress transcription factor B-2c
	OS10G0419300	up	Similar to Heat shock transcription factor 31
	OS02G0232000	down	Similar to Heat shock transcription factor 29
	OS03G0854500	down	Similar to Heat shock transcription factor 31
	OS04G0568700	down	Similar to Heat stress transcription factor Spl7
	OS06G0553100	down	Similar to Heat stress transcription factor C-2b
	OS07G0178600	down	Similar to Heat shock transcription factor 29
	OS07G0640900	down	Similar to Isoform 2 of Heat stress transcription factor B-4b
	OS09G0456800	down	Similar to Heat stress transcription factor Spl7
HRGD1vsHRGD0	OS08G0546800	up	Similar to Heat stress transcription factor B-2b
	OS09G0456800	up	Similar to Heat stress transcription factor Spl7
LRGD1vsLRGD0	OS03G0745000	down	Similar to Heat stress transcription factor A-2a
	OS08G0546800	down	Similar to Heat stress transcription factor B-2b
	OS09G0526600	down	Similar to Isoform 2 of Heat stress transcription factor B-2c
	OS10G0419300	down	Similar to Heat shock transcription factor 31

### Terms of gene ontology (GO) associated with glume-unclosing

**Figure 5:**
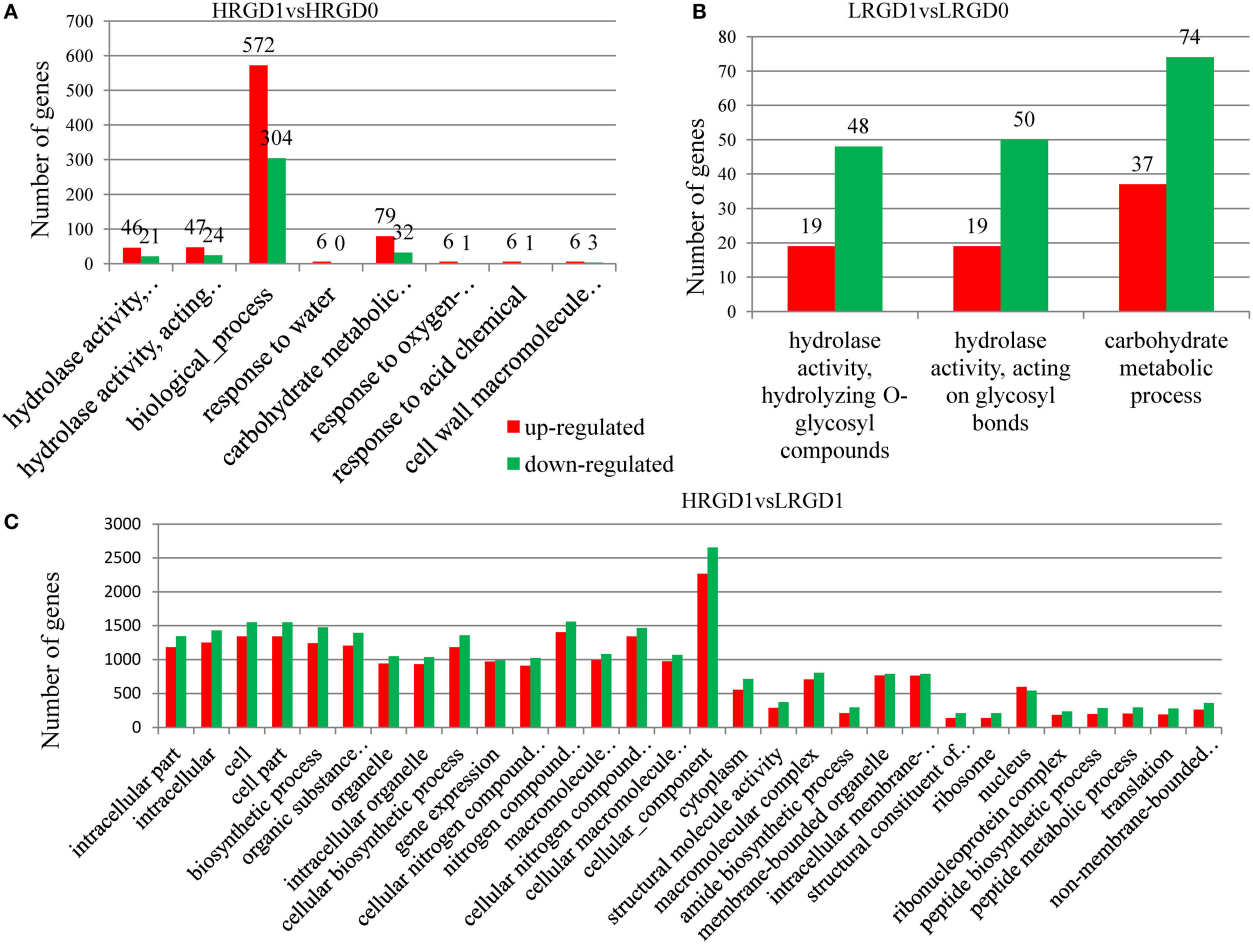
**Enrichment of up-and down-regulated DEGs in different functional categories in different sample pairs. (A)** GO terms listed (*p* < 0.05) in HRGD1vsHRGD0. **(B)** GO terms listed (*p* < 0.05) in LRGD1vsLRGD0. **(C)** Top 30 GO terms listed (*p* < 0.05) in HRGD1vsLRGD1.

The analysis of Gene Ontology (GO) was conducted to explore the biological processes related to glume-opening. In HRGD1vsHRGD0, the DEGs are significantly enriched in 8 GO terms (FDR < 0.05), including hydrolase activity that hydrolyzes O-glycosyl compounds (GO:0004553) (67 genes), hydrolase activity that acts on glycosyl bonds (GO:0016798) (71 genes), biological process (GO:0008150) (876 genes), response to water (GO:0009415) (6 genes), carbohydrate metabolic process (GO:0005975) (111 genes), response to oxygen-containing compound (GO:1901700) (7 genes), response to acid chemical (GO:000110) (7 genes), and cell wall macromolecule catabolic process (GO:0016998) (9 genes) (Figure 5A). In LRGD1vsLRGD0, the DEGs are grouped into 3 GO terms (FDR < 0.05), including hydrolase activity that hydrolyzes O-glycosyl compounds (GO:0004553) (67 genes), hydrolase activity that acts on glycosyl bonds (GO:0016798) (69 genes), and carbohydrate metabolic process (GO:0005975) (111genes) (Figure 5B). In HRGD0vsLRGD0, the differentially expressed genes are not significantly enriched in any terms (corrected *P* < 0.05). In HRGD1vsLRGD1, the differentially expressed genes are remarkably enriched in 119 GO terms (corrected *P* < 0.05) (Figure 5C).

**Figure 6:**
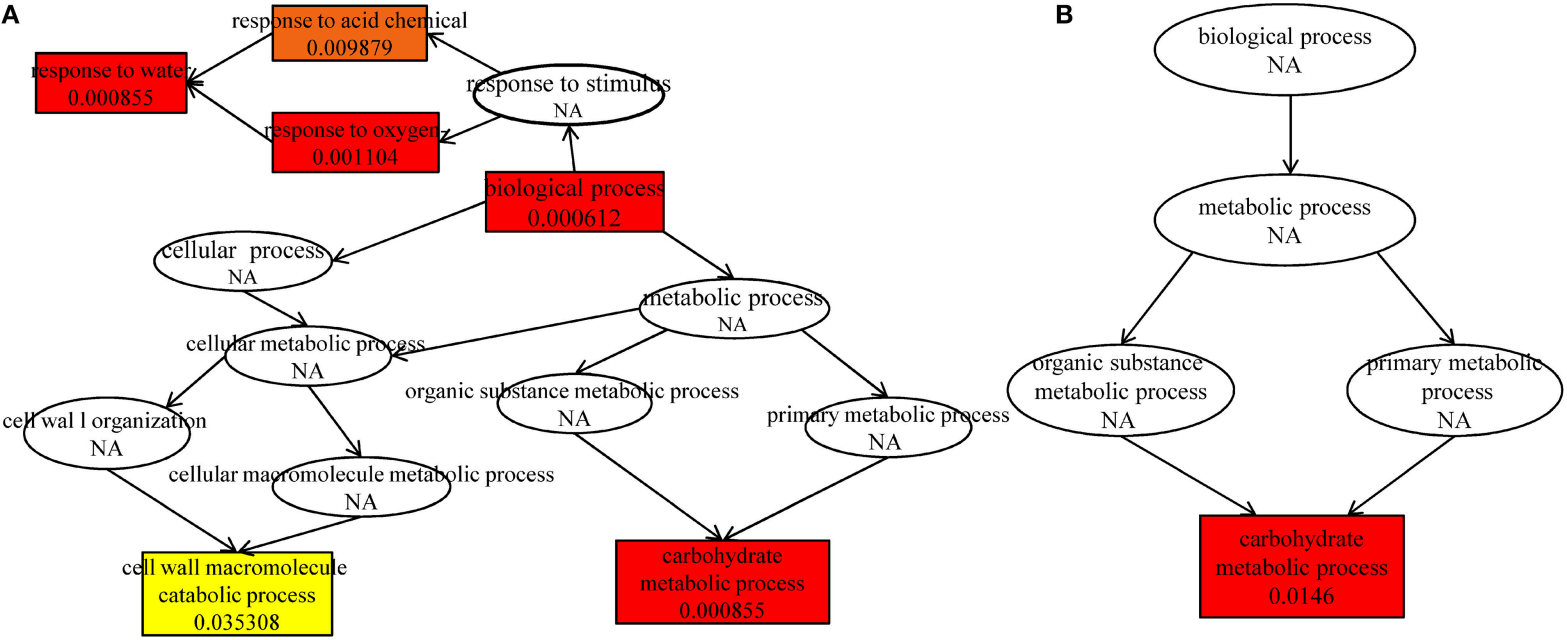
**The enriched biological processes under different temperatures**. The different color frames indicate the extent of significance. Yellow: significant; Red: extremely significant. **(A)** In HRGD1vsHRGD0. **(B)** In LRGD1vsLRGD0.

Further analysis indicated that up-regulated DEGs were significantly enriched in the terms of biological process, response to water, carbohydrate metabolic process, response to oxygen-containing compound, response to acid chemical in HRGD1vsHRGD0 (Figure 6A), and the down-regulated DEGs were remarkably grouped into the term of carbohydrate metabolic process in LRGD1vsLRGD0 (Figure 6B), indicating that carbohydrate metabolic process may play a pivotal role in glume-unclosing after anthesis under high temperature.

### Identification of DEGs related to glume-unclosing

Rice floret opening depends on relaxation of cell wall of lodicules and influx of water (Wang and Gu, 2012). We investigated the DEGs enriched in the terms of carbohydrate metabolic process (GO:0005975), response to water (GO:0009415), and cell wall macromolecule catabolic process (GO:0016998) in HRGD1vsHRGD and LRGD1vsLRGD0.

The term of carbohydrate metabolic process was enriched in HRGD1vsHRGD0 and LRGD1vsLRGD0. Seventeen up- and 17 down-regulated DEGs were shared in HRGD1vsHRGD and LRGD1vsLRGD0 (Table S5). Three genes (Os03G0277300, Os01G0840100, and Os06G0696600) were up-regulated in HRGD1vsHRGD0 whereas down-regulated in LRGD1vsLRGD0. Os03G0277300 and Os01G0840100 encode heat shock protein Hsp70 family proteins, and Os06G0696600 (*OsXTH12*) encodes a protein similar to xyloglucan endo-transglycosylase. We conclude that these genes may play an important role in regulating glume-unclosing after anthesis under high temperature.

In HRGD1vsHRGD0, 6 up-regulated genes were enriched in the term of response to water (Table 3). We also investigated their expression in LRGD1vsLRGD0 and found that two genes (Os11G0454200 and Os11G0454300) were significantly down-regulated and the other 4 genes (Os11G0453900, Os11G0451700, Os11G0454000, and Os01G0702500) were not differentially expressed. These results indicate that Os11G0454200 and Os11G0454300 may regulate the flow of water in the course of floret opening.

**Table 3:** **The DEGs enriched in the term of response to water in HRGD1 vs HRGD0 and LRGD1 vs LRGD0**.

**Gene name**	**HRGD1 vs HRGD0**	**LRGD1 vs LRGD0**	**Description**
OS11G0453900	up	N	RAB(RESPONSIVE TO ABA) GENE 16D, Dehydrin Rab16D
OS11G0451700	up	N	Similar to Dehydrin DHN1 (M3) (RAB-17 protein)
OS11G0454000	up	N	RAB(RESPONSIVE TO ABA) GENE 16C, Dehydrin Rab16C
OS01G0702500	up	N	Similar to Dehydrin Rab25, Responsive to abscisic acid 25
OS11G0454200	up	down	Dehydrin Rab16B, RAB(RESPONSIVE TO ABA) GENE 16B
OS11G0454300	up	down	Similar to Water-stress inducible protein RAB21,RAB(responsive to ABA) gene 16A

*N, no differential expression*.

Nine genes were enriched in the term of cell wall macromolecule catabolic process in HRGD1vsHRGD0 and most of them are associated with glycoside hydrolase activity (Table 4). Five genes in HRGD1vsHRGD0 showed the same expression pattern as in LRGD1vsLRGD0. And the other 4 up-regulated genes (Os06G0726200, Os06G0726100, Os10G0542900, and Os02G0605900) in HRGD1vsHRGD0 were not differentially expressed in LRGD1vsLRGD0, indicating that these 5 genes may be involved in cell wall loosening before cell expansion in rice lodicules during anthesis whereas the other 4 genes may only affect cell wall macromolecule hydrolysis in rice lodicules under high temperature.

**Table 4:** **The DEGs enriched in the term of cell wall macromolecule catabolic process in HRGD1 vs HRGD0 and LRGD1 vs LRGD0**.

**Genes**	**HRGD1 vs HRGD0**	**LRGD1 vs LRGD0**	**Description**
OS11G0557500	up	up	Protein kinase, core domain containing protein
OS05G0399300	up	up	Similar to Chitinase, Glycoside hydrolase, family 19
OS06G0726200	up	N	Similar to Chitinase 1, Glycoside hydrolase, family 19
OS06G0726100	up	N	Similar to Seed chitinase-c, Glycoside hydrolase, family 19
OS10G0542900	up	N	Similar to chitinase, Glycoside hydrolase, family 19
OS02G0605900	up	N	Similar to Chitinase (EC 3.2.1.14) A, Glycoside hydrolase, family 19
OS05G0399700	down	down	Chitinase (EC 3.2.1.14), Glycoside hydrolase, family 19
OS05G0399400	down	down	Chitinase 9, Glycoside hydrolase, family 19
OS09G0452200	down	down	Peptidoglycan and chitin perception in innate immunity

*N, No differential expression*.

### Identification of genes specific to glume-unclosing dependent on high temperature

**Figure 7:**
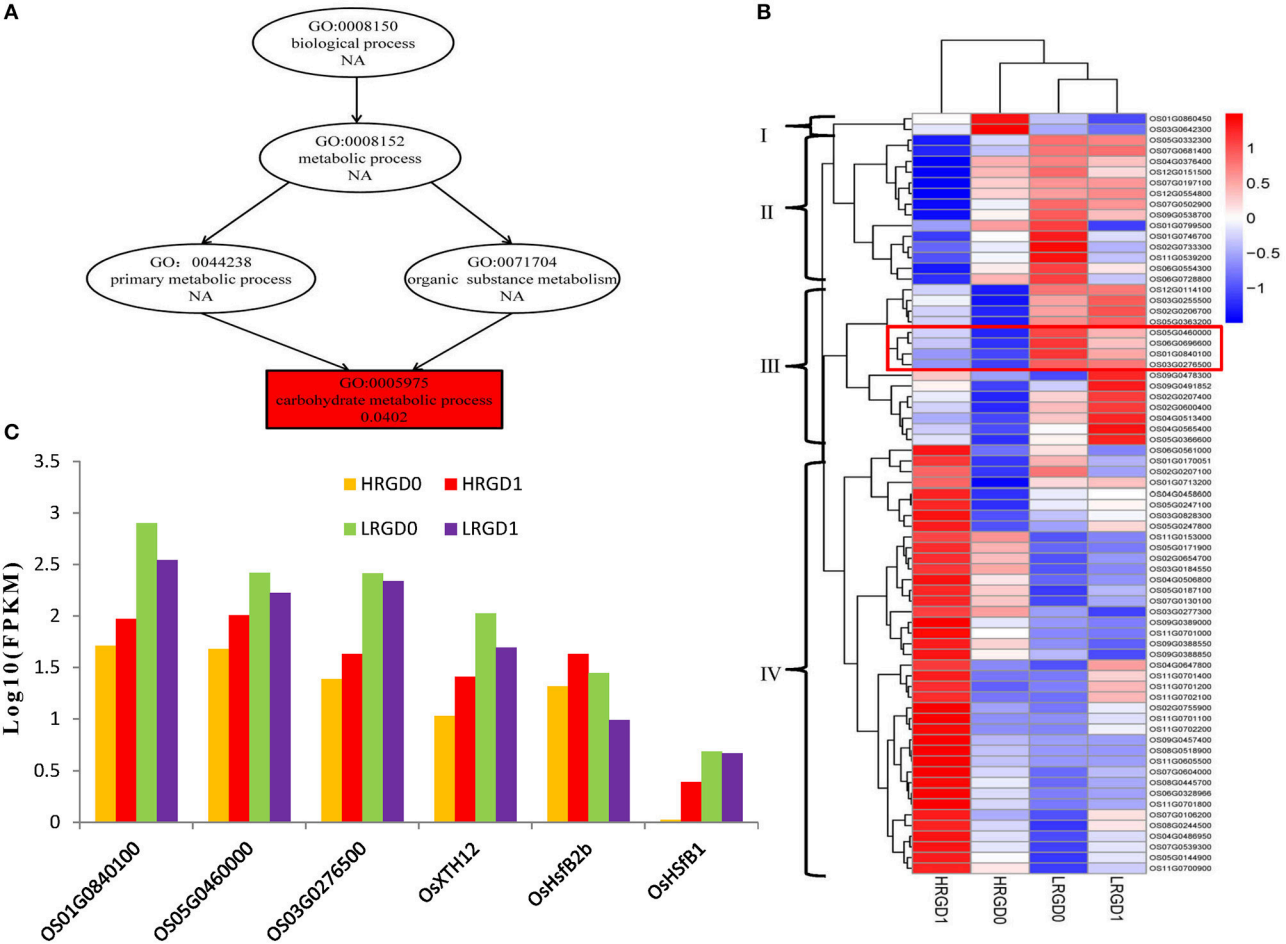
**The remarkably enriched DEGs shared in HRGD1vsHRGD0 and HRGD1vsLRGD1. (A)** The enriched biological process in HRGD1vsHRGD0 and HRGD1vsLRGD1. **(B)** Heat map of scaled FPKM values of 71 genes enriched in the carbohydrate metabolic process in samples HRGD1, HRGD0, LRGD1, and LRGD0. **(C)** The expression patterns of six genes including three HSP70 genes (Os01G0840100, Os05G0460000, and Os03G0276500), two HSF genes *OsHsfB1* (OS09G0456800) and *OsHsfB2b* (OS08G0546800) and one xyloglucan endo-transglycosylase–like gene *OsXTH12* (Os06G0696600).

The 871 genes shared in HRGD1vsHRGD0 and HRGD1vsLRGD1, including two class B HSF genes *OsHsfB2b* (Os08G0546800) and *OsHsfB1* (Os09G0456800) were considered to be specifically related to rice glume-unclosing after anthesis under high temperature. GO analysis indicated that only the term of carbohydrate metabolic process, containing 71 DEGs, was significantly enriched (FDR < 0.05) (Figure 7A). In the 71 genes, 4 genes (Os03G0277300, Os01G0840100, Os05G0460000, and Os03G0276500) belong to the heat shock protein 70 (HSP70) family; in addition, some genes encode glycoside hydrolase or glycosyl transferase, implying that these heat shock proteins may be involved in regulation of carbohydrate metabolism. Based on the expression levels in the four samples, these 71 genes were further divided into four groups (Figure 7B). We found that three heat shock protein genes (Os01G0840100, Os05G0460000, and Os03G0276500) and *OsXTH12* (Os06G0696600, encoding a protein, similar to xyloglucan endo-transglycosylase) were clustered into a subfamily of Group III, indicating that they have similar expression patterns. We also compared the expression pattern of the two HSF genes, the three HSP genes and *OsXTH12* at the stages of pre- and post-anthesis under high and low temperature (Figure 7C), and found that *OsHsfB2b, OsXTH12*, and HSP genes Os01G0840100, Os05G0460000, and Os03G0276500 showed similar expression pattern. Namely, their expression was down-regulated at the post-anthesis stage under low temperature. Whereas, their expression was up-regulated at the post-anthesis stage under high temperature. Another HSF gene *OsHsfB1* didn't differentially express under low temperature, and its expression was up-regulated at the stage of post-anthesis under high temperature, indicating *OsHsfB1* may be house-keeping gene and was specifically up-regulated at post-anthesis under high temperature. Hence, *OsHsfB1* and *OsXTH12* are good candidate genes controlling glume-unclosing under high temperature in RGD-7S.

## Discussion

Factors influencing flower opening include changes to carbohydrate metabolism (particularly breakdown of storage carbohydrates to soluble, osmotically active sugars), water flow affecting cell turgor, and cell wall metabolism (van Doorn and van Meeteren, 2003). In this study, we found that several biological processes related to carbohydrate metabolism, response to water and cell wall macromolecular metabolism were involved in rice glume-unclosing after anthesis dependent on high temperature in rice TSGM line RGD-7S.

### Carbohydrate metabolism and glume-unclosing

Both up-regulated genes in HRGD1vsHRGD0 and down-regulated genes in LRGD1vsLRGD0 were significantly enriched in carbohydrate metabolic process. The 71 DEGs shared in HRGD1vsHRGD0 and HRGD1vsLRGD1 specifically for the phenotype of glume-unclosing after anthesis were enriched only in carbohydrate metabolic process. These results indicate that carbohydrate metabolism may play a key role in the unclosing of rice glumes under high temperature in RGD-7S. Several recent studies also reported that carbohydrate metabolism played an important role in the coordination of developmental transitions and flower opening (Cho et al., 2006; Norikoshi et al., 2013; Wahl et al., 2013; Yang et al., 2013; Stitz et al., 2014).

The 71 DEGs enriched in carbohydrate metabolic process include four heat shock protein genes belonging to the HSP70 family, suggesting they may be involved in carbohydrate metabolism. Carbohydrate metabolism is influenced by heat shock in Arabidopsis (Kaplan et al., 2004). Heat shock proteins as molecular chaperones are involved in signaling, translation, host-defense mechanisms, carbohydrate metabolism and amino acid metabolism (Usman et al., 2014). Besides its general chaperone functions, HSP70 also regulates the expression of other stress associated genes (Montero-Barrientos et al., 2010). Wang et al. (2014) reported that HSP70 genes could play an important role in stimulating carbohydrate metabolism by regulating the activity of certain key enzymes in rice. In addition, several HSP70s also play a crucial role in housekeeping activities under favorable conditions (Tompa and Kovacs, 2010). Two (Os03G0277300 and Os01G0840100) of the four HSP70 genes were up-regulated under high temperature (in HRGD1vsHRGD0) and down-regulated under low temperature (in LRGD1vsLRGD0), indicating that they may have housekeeping activities and their expression is also induced by high temperature.

Several overlapped DEGs have glycosyl hydrolase (GH) activity and are up-regulated under high temperature in this study. Arabidopsis GHs are predicted to be localized in the cell wall and likely participate in cell wall remodeling (Showalter, 1993; Minic, 2008). Most plant GHs were reported to participate in the metabolism of cell wall polysaccharides (Cosgrove, 2005; Minic and Jouanin, 2006). Additionally, different stresses such as heat, salt, and dehydration can induce expression of GH genes (Sharma et al., 2013). Hence, under high temperature, up-regulation of these glycosyl hydrolase-encoding DEGs could promote degradation of polysaccharides in cell walls of rice lodicules and accumulate soluble monosaccharides, resulting in reduction of osmotic pressure.

We detected that *OsXTH12* (Os06G0696600), a xyloglucan endo-transglycosylase–like gene, expressed much higher at the stage of pre-anthesis than post-anthesis under low temperature, and much higher after flowering than before flowering under high temperature. These results indicate that the expression pattern of *OsXTH12* at the stage of rice floret opening is hypersensitive to and easily affected by temperature changes. Most of the 29 annotated rice xyloglucan endo-transglycosylase/hydrolase (XTH) genes show tissue-specific and growth stage-dependent expression patterns; *OsXTH12* had low levels of expression in leaf, shoot and root (Yokoyama et al., 2004). These *XTH*-encoded proteins potentially have xyloglucan endo-transglycosylase (XET) and xyloglucan endo-hydrolase (XEH) activities yielding irreversible chain shortening of xyloglucans (Eklöf and Brumer, 2010). XETs can cause transient matrix cleavage without hydrolysis, providing a potential molecular mechanism for controllable turgor-driven wall expansion (Rose et al., 2002). XTHs are involved in diverse physiological processes such as seed germination, organogenesis, cell expansion, and fruit ripening (Yokoyama et al., 2004). In rose, the up-regulated expression of 4 *XTH* genes (*RbXTH3, RbXTH5, RbXTH6, and RbXTH12*) was associated with anthesis and petal movement during flower opening (Singh et al., 2013). We conducted BLAST analysis among the protein sequences of *OsXTH12* and these 4 *RbXTHs* and found that the sequence identity was approximately 60% (Figure S1), indicating they that may share a similar role in regulation of anthesis. The expression of Arabidopsis *TCH4* showed an unusual temperature dependence (Purugganan et al., 1997). The protein sequences of OsXTH12 and Arabidopsis TCH4 also share more than 70% identity (Figure S2). We hypothesize that *OsXTH12* may be specifically expressed in rice floral organs and its expression may be affected by temperature and developmental phases, making it a good candidate gene causing rice glume-unclosing after anthesis under high temperature.

### Response to water and glume-unclosing

Under high temperature condition, 6 up-regulated genes encoding dehydrin RAB21/16A, RAB16B, RAB16C, RAB16D, RAB17, and RAB25 proteins were enriched in the biological process of response to water, and two of them were down-regulated in LRGD1vsLRGD0. These results suggest that these genes may participate in the inflow and outflow of water in rice lodicules. Dehydrins are a class of hydrophilic, thermostable stress proteins with a high number of charged amino acids (Yang et al., 2012). RAB21/16A, RAB16B, RAB16C, RAB16D, RAB25 were identified as osmotic stress-responsive dehydrins (Lee et al., 2005; Kumar et al., 2014). It was hypothesized that these dehydrins may carry out their function through membrane stabilization by acting as chaperones to prevent the aggregation and/or inactivation of proteins under dehydration or high temperature conditions (Kovacs et al., 2008; Peng et al., 2008; Brini et al., 2011). The closing of florets was caused by the rupture of the tonoplast and cell autolysis in lodicules and the outflow of water (Wang and Gu, 2012). It is possible that up-regulation of these 6 genes leads to stabilization of the tonoplast and maintenance of the swelling status of lodicules, causing the glume-unclosing under high temperature, and that down-regulation of two of them destabilizes the tonoplast triggering autolysis of lodicules, resulting in the closing of rice glumes under low temperature.

### Cell wall macromolecular metabolism and glume-unclosing

In our study, seven of 9 genes related to cell wall macromolecular metabolism under high temperature encode a protein similar to chitinase. Recent reports indicated that plant chitinases play an important role in developmental processes including flower development and seed development by generating or degrading signal molecules (Van Damme et al., 1999; Takakura et al., 2000; Mokshina et al., 2014). Several chitinase genes predominantly expressed in flowers have been identified in potato (Wemmer et al., 1994), *Brassica napus* (Hamel and Bellemare, 1995), tomato (Harikrishna et al., 1996), and rice (Takakura et al., 2000). These genes were found highly expressed in the floral organs while only expressed at low levels in vegetable organs. Rice chitinase gene *OsChia1;175* (Os05G0399700) was expressed maximally in three floral organs (lodicules, stigmas, and pistils) at the heading to flowering stages and its transcripts were not detected in other organs (Takakura et al., 2000). Our results also showed that *OsChia1;175* was down-regulated at the post-anthesis stage compared with the heading to flowering stages under both high and low temperatures (Table S5), which suggests that this gene may play an important role in cell expansion of rice lodicules at the heading to flowering stages.

Several reports indicate that heat shock enhances the expression of plant chitinase genes (Hong et al., 2003; Kwon et al., 2007; Takenaka et al., 2009; Hermans et al., 2010). We also detected up-regulation of four chitinase-like genes belonging to glycoside hydrolase family 19 at the post-anthesis stage only under high temperature, which is consistent with the previous conclusion that all of the identified stress-responsive chitinase genes belong to the GH-19 family (class I and class II) (Takenaka et al., 2009).

### Heat shock response and glume-unclosing

Plants respond to heat shock by perception of the signal at the membrane (Saidi et al., 2009) activating HSF gene to enhance the expression of HSP genes (Chauha et al., 2011). The expression of HSP genes were regulated by HSFs through recognizing the heat shock elements present in the promoter region (Nover et al., 2001). HSPs as molecular chaperones play a key role in the folding, intracellular distribution, and degradation of proteins against stress damage (Guo et al., 2008). In this study, we detected a group of heat shock genes including HSF genes, HSP genes and other inducible genes in the panicles at the stages of pre- and post-anthesis, suggesting the diversity of HSFs provides redundancy and specialization of stress signals in plants (Morimoto, 1998). The HSF gene *OsHsfB2b*, the xyloglucan endo-transglycosylase–like gene *OsXTH12*, and three HSP genes showed similar expression patterns. The expression of another HSF gene *OsHsfB1* appeared to be specifically up-regulated at the stage of post-anthesis under high temperature. We found that the two detected HSF genes *OsHsfB2b* and *OsHsfB1* at post-anthesis under high temperature belonged to class B HSF. In plants, class B HSFs is required to act as repressors of the expression of the HS-inducible HSF genes (Ikeda et al., 2011).

In a variety of plant species including rice, HsfB1 contained the 11 amino acid (GEGLKLFGVWL) at the C terminus as a repression domain, designated the B3 repression domain (BRD), required for the HsfB1-mediated repression of the expression of downstream genes (Ikeda and Ohme-Takagi, 2009; Ikeda et al., 2011). In Arabidopsis, HsfB1 and HsfB2b are localized in the nucleus and directly repress the expression of several HSF genes (*HsfA2, HsfA7a, HsfB1*, and *HsfB2b*) and some HSP genes (Scharf et al., 1998; Heerklotz et al., 2001; Kotak et al., 2004; Ikeda et al., 2011). *HsfB1* was expressed under normal conditions and was also not up-regulated at 28°C, whereas the expression of *HsfB2b* was up-regulated at 28°C (Busch et al., 2005; Schramm et al., 2008). However, the expression of *HsfB1* and *HsfB2b* was up-regulated at 32°C (Ikeda et al., 2011). In our study, the expression patterns of two class B HSF genes *OsHsfB1* and *OsHsfB2b* at pre- and post-anthesis in rice panicles are similar to *HsfB1* and *HsfB2b* in Arabidopsis. Hence, we hypothesize that *OsHsfB1* may repress the expression of *OsHsfB2b, OsXTH12*, and three HSP genes Os01G0840100, Os05G0460000, and Os03G0276500 at post-anthesis under low temperature, whereas the up-regulated *OsHsfB1* enhances their expression, especially *OsXTH12* at post-anthesis under high temperature to induce rapid matrix cleavage, and loosen cell wall of lodicules accompanying water uptake, causing glume-unclosing.

## Author contributions

CF, writing, analyzing the data of RNA seq and designing the experiment; FW, revising the manuscript; WL and DL, carrying the real time PCR analysis; JL, MZ, YL, ZL, HH, XZ, and XM, investigating the phenotype of glume-cunclosing and treating the plants.

### Conflict of interest statement

The authors declare that the research was conducted in the absence of any commercial or financial relationships that could be construed as a potential conflict of interest.

## Supplementary material

Figure S1 **BLAST analysis of the putative protein sequences of four rose XTH genes and *OsXTH12* (Os06G0696600)**.

Figure S2 **BLAST analysis of the putative protein sequences between OsXTH12 and Arabidopsis TCH4**.

Table S1 **The primer sequences of 31 selected genes used in real time PCR**.

Table S2 **Quality of the RNA sequencing data**.

Table S3 **Information of reads aligned to the reference sequence**.

Table S4 **Pearson correlation between RNA-seq data from different samples**.

Table S5 **The DEGs enriched in the biological process of carbohydrate metabolism in HRGD1vsHRGD0 and LRGD1vsLRGD0**.
