# Supplementary figures and images for: Transcriptomic Analysis Reveals New Insights into High-Temperature-Dependent Glume-Unclosing in an Elite Rice Male Sterile Line
Source: Front Plant Sci. 2017 Feb 14;8:112. doi: 10.3389/fpls.2017.00112 (PMC5306291; doi:10.3389/fpls.2017.00112)

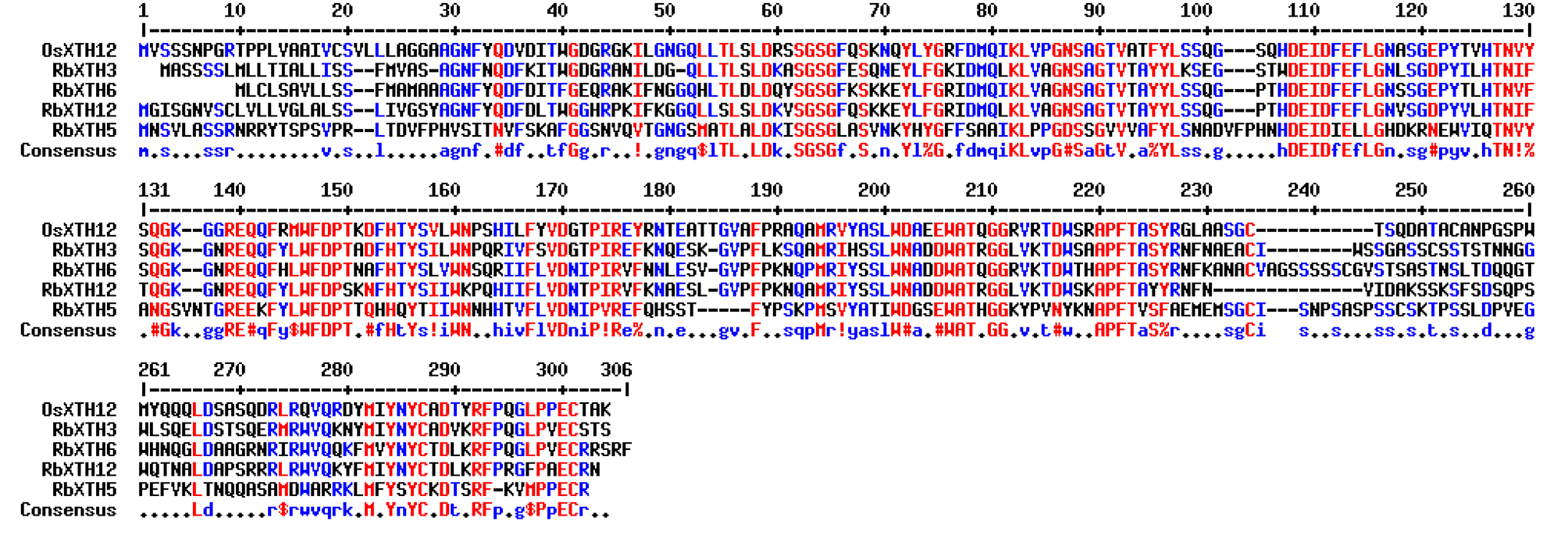

Supplement: Figure S1 — BLAST analysis of the putative protein sequences of four rose XTH genes and OsXTH12 (Os06G0696600). [file Image1.TIF]

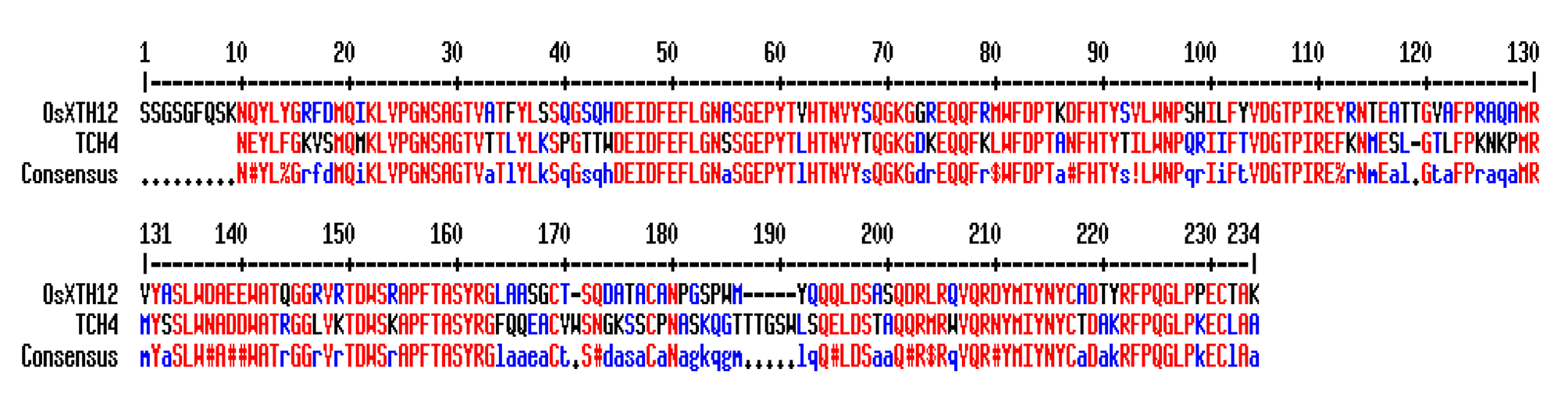

Supplement: Figure S2 — BLAST analysis of the putative protein sequences between OsXTH12 and Arabidopsis TCH4. [file Image2.TIF]
