# Supplementary material for: Transcriptomic Analysis Reveals New Insights into High-Temperature-Dependent Glume-Unclosing in an Elite Rice Male Sterile Line
Source: Front Plant Sci. 2017 Feb 14;8:112. doi: 10.3389/fpls.2017.00112 (PMC5306291; doi:10.3389/fpls.2017.00112)
Supplement: Table S1 — The primer sequences of 31 selected genes used in real time PCR. [file Table1.DOCX]

Table S1The primer sequences of 31 selected genes used in real time PCR

| Gene ID | Forward Primer | Reverse Primer |
| --- | --- | --- |
| OS11G0700900 | CTTGACGGGGAAGAACGACA | GACGTATCGGCCGTAGTGAG |
| OS09G0389000 | TCGTTCAAGGCTGCATCAGT | CTAAAGCGAGTAGCAGCCGA |
| OS11G0702100 | CGCCGACAAAGCCTACGATA | GCTCCGATGAAGCAGCAATG |
| OS12G0151500 | TCCACTACTCGTCGGGGATG | GTGTGGTAATGGTGCTTGGC |
| OS03G0642300 | GCTCGAGTACCTGCACATGA | GACAGGTCGAAGTCGGTGAG |
| OS05G0332300 | AGCCGAAAAGGGTATGACGG | GCTGGGCATTTGAACTCTGC |
| OS02G0733300 | CAGCGAGTCCATCAACGAGT | TGACACGGAACGACTGGAAG |
| OS03G0277300 | GAGGATGCCATCAAGTGGCT | CCGCCCTGGTACATCTTTGA |
| OS01G0840100 | TATGTCGCGTTCACCGACTC | TCATGTCACTCTGCACGGAC |
| OS01G0746700 | ACCTCCGATCCATCTGGTGA | TTCTTGAGAACTCGTCGGGC |
| OS11G0701800 | CGCAACCTCTACGCCTACAA | GCCTTCTTCATCCTCGGGTC |
| OS12G0554800 | CCATCCTTCGCTGTTCCAGT | TCCATGAGCCTCAAAGCTGG |
| Os01g0713200 | AGTCGCCATCGTACAGCATT | CACGGCACTATGCTTGCTTC |
| OS11G0701000 | GTGACGGGGAAGAACGACAT | CTGTTCCAGAGCGTCACTCC |
| OS03G0828300 | GCCTCACCAACAACTTCGAC | TGTGACGTACCTTGACGTGG |
| OS08G0518900 | CGCCGACAACCTCTACAACT | ACCGCGGTCAATGAAGAAGT |
| OS07G0106200 | CATGCTCATCGTCGGTCGTA | CATCAGCTGGAACCCGATGT |
| OS09G0538700 | CCTCCCGAGACCAACAACAG | CTACCTTTGCTGTACCCGCT |
| OS01G0860450 | GGATCAGGAATCACGACCCG | CATCTCGTACCCCAAGTCGG |
| OS05G0460000 | CCATCCCGACCAAGAAGGAG | AACTTGCCGAGGAGGTTGTT |
| Os03g0276500 | ATGATCGGCGTGCAGTACAA | GACGGTGCTTCCGAGATAGG |
| OS08G0244500 | CAGACCGGCTCGTTCTTCAT | TCGAGCACACCAGCATTAGG |
| OS11G0701200 | CTGCCGCGTAAGGTTCTCTT | GTAGCCGGTCTTCTTGTCGT |
| OS11G0701400 | ATACGATCGATGGCGTTCCG | ATTACGGCGGTCTGGTCTTC |
| OS11G0702200 | CTACACCACCGTCGTCATCT | GAGGACGGTGACGTTCTTGG |
| OS04G0376400 | CCCGTAGGAGCCAAGAATCG | GGCGTAGTAGTAGAGGTGCG |
| OS08G0445700 | GCCGCATCTCCACTATCTCC | AAGTGGGTGTTGACGGTGAG |
| OS04G0513400 | ACGCTTGACACTTACGAGGG | TGGCTTCTGGGACGACAAAG |
| OS11G0701100 | ACAACAAGTACTACCGGGGC | TCGTCACCGAACATCCTCAC |
| OS04G0486950 | CAGCACTTCATGCGCTCCTA | GTCCTTGATCGGGATCTGCG |
| OS05G0247100 | GGGACAAGAAGACCGGATGG | AGCTCGTGCATGAATCCAGT |
| *Osactin1* | GACCCAGATCATGTTTGAGACCT | CAGTGTGGCTGACACCATCAC |
