# Supplementary material for: Transcriptomic Analysis Reveals New Insights into High-Temperature-Dependent Glume-Unclosing in an Elite Rice Male Sterile Line
Source: Front Plant Sci. 2017 Feb 14;8:112. doi: 10.3389/fpls.2017.00112 (PMC5306291; doi:10.3389/fpls.2017.00112)
Supplement: Table S2 — Quality of the RNA sequencing data. [file Table2.DOCX]

Table S2. Quality of the RNA sequencing data

| Sample name | Raw reads | Clean reads | Clean bases | Error rate(%) | Q20(%) | Q30(%) | GC content(%) |
| --- | --- | --- | --- | --- | --- | --- | --- |
| HRGD0_1 | 54,108,298 | 50,064,604 | 7.51G | 0.02 | 96.02 | 90.9 | 54.26 |
| HRGD0_2 | 49,508,210 | 45,144,260 | 6.77G | 0.02 | 96.36 | 91.6 | 55.77 |
| HRGD0_3 | 58,389,834 | 53,821,062 | 8.07G | 0.02 | 96.35 | 91.54 | 55.14 |
| HRGD1_1 | 51,958,532 | 48,088,992 | 7.21G | 0.02 | 96.35 | 91.56 | 55.53 |
| HRGD1_2 | 50,296,520 | 46,676,298 | 7.0G | 0.02 | 96.4 | 91.67 | 54.01 |
| HRGD1_3 | 44,355,422 | 41,062,990 | 6.16G | 0.02 | 96.37 | 91.58 | 56.02 |
| LRGD0_1 | 51,589,058 | 47,765,702 | 7.16G | 0.02 | 96.42 | 91.68 | 55.89 |
| LRGD0_2 | 58,957,104 | 54,020,206 | 8.1G | 0.02 | 96.46 | 91.76 | 54.4 |
| LRGD0_3 | 59,221,448 | 54,998,760 | 8.25G | 0.02 | 96.2 | 91.24 | 54.37 |
| LRGD1_1 | 56,875,164 | 52,814,628 | 7.92G | 0.02 | 96.26 | 91.36 | 53.73 |
| LRGD1_2 | 53,166,386 | 49,340,840 | 7.4G | 0.02 | 96.26 | 91.33 | 53.11 |
| LRGD1_3 | 55,639,434 | 51,751,694 | 7.76G | 0.02 | 96.34 | 91.48 | 53.56 |
