# Supplementary material for: Transcriptomic Analysis Reveals New Insights into High-Temperature-Dependent Glume-Unclosing in an Elite Rice Male Sterile Line
Source: Front Plant Sci. 2017 Feb 14;8:112. doi: 10.3389/fpls.2017.00112 (PMC5306291; doi:10.3389/fpls.2017.00112)
Supplement: Table S3 — Information of reads aligned to the reference sequence. [file Table3.DOCX]

Table S3. Information of reads aligned to the reference sequence

| Sample_name | Total reads | Total mapped | Multiple mapped | Uniquely mapped | Non-splice reads | Splice reads |
| --- | --- | --- | --- | --- | --- | --- |
| HRGD0_1 | 50064604 | 37917148 (75.74%) | 684560 (1.37%) | 37232588 (74.37%) | 23712409 (47.36%) | 13520179 (27.01%) |
| HRGD0_2 | 45144260 | 32993376 (73.08%) | 605337 (1.34%) | 32388039 (71.74%) | 23226684 (51.45%) | 9161355 (20.29%) |
| HRGD0_3 | 53821062 | 40524294 (75.29%) | 1206881 (2.24%) | 39317413 (73.05%) | 26027250 (48.36%) | 13290163 (24.69%) |
| HRGD1_1 | 48088992 | 36387074 (75.67%) | 645960 (1.34%) | 35741114 (74.32%) | 23012140 (47.85%) | 12728974 (26.47%) |
| HRGD1_2 | 46676298 | 35579356 (76.23%) | 633121 (1.36%) | 34946235 (74.87%) | 21938746 (47%) | 13007489 (27.87%) |
| HRGD1_3 | 41062990 | 31308385 (76.24%) | 556120 (1.35%) | 30752265 (74.89%) | 19880796 (48.42%) | 10871469 (26.48%) |
| LRGD0_1 | 47765702 | 36868079 (77.19%) | 563071 (1.18%) | 36305008 (76.01%) | 24786944 (51.89%) | 11518064 (24.11%) |
| LRGD0_2 | 54020206 | 41244551 (76.35%) | 1121858 (2.08%) | 40122693 (74.27%) | 29086040 (53.84%) | 11036653 (20.43%) |
| LRGD0_3 | 54998760 | 43295061 (78.72%) | 888997 (1.62%) | 42406064 (77.1%) | 26952061 (49%) | 15454003 (28.1%) |
| LRGD1_1 | 52814628 | 41232475 (78.07%) | 971115 (1.84%) | 40261360 (76.23%) | 25413282 (48.12%) | 14848078 (28.11%) |
| LRGD1_2 | 49340840 | 37666609 (76.34%) | 906171 (1.84%) | 36760438 (74.5%) | 22880067 (46.37%) | 13880371 (28.13%) |
| LRGD1_3 | 51751694 | 40682696 (78.61%) | 661179 (1.28%) | 40021517 (77.33%) | 24807353 (47.94%) | 15214164 (29.4%) |
