# Supplementary material for: Transcriptomic Analysis Reveals New Insights into High-Temperature-Dependent Glume-Unclosing in an Elite Rice Male Sterile Line
Source: Front Plant Sci. 2017 Feb 14;8:112. doi: 10.3389/fpls.2017.00112 (PMC5306291; doi:10.3389/fpls.2017.00112)
Supplement: Table S4 — Pearson correlation between RNA-seq data from different samples. [file Table4.DOCX]

Table S4 Pearson correlation between RNA-seq data from different samples

| R^2 | HRGD0_1 | HRGD0_2 | HRGD0_3 | HRGD1_1 | HRGD1_2 | HRGD1_3 | LRGD0_1 | LRGD0_2 | LRGD0_3 | LRGD1_1 | LRGD1_2 | LRGD1_3 |
| --- | --- | --- | --- | --- | --- | --- | --- | --- | --- | --- | --- | --- |
| HRGD0_1 | 1 | 0.963 | 0.979 | 0.966 | 0.969 | 0.958 | 0.905 | 0.885 | 0.91 | 0.904 | 0.901 | 0.905 |
| HRGD0_2 | 0.963 | 1 | 0.972 | 0.95 | 0.946 | 0.947 | 0.906 | 0.906 | 0.89 | 0.89 | 0.883 | 0.89 |
| HRGD0_3 | 0.979 | 0.972 | 1 | 0.969 | 0.968 | 0.961 | 0.909 | 0.896 | 0.907 | 0.904 | 0.901 | 0.904 |
| HRGD1_1 | 0.966 | 0.95 | 0.969 | 1 | 0.982 | 0.98 | 0.897 | 0.877 | 0.899 | 0.906 | 0.908 | 0.91 |
| HRGD1_2 | 0.969 | 0.946 | 0.968 | 0.982 | 1 | 0.975 | 0.894 | 0.875 | 0.899 | 0.905 | 0.906 | 0.909 |
| HRGD1_3 | 0.958 | 0.947 | 0.961 | 0.98 | 0.975 | 1 | 0.883 | 0.865 | 0.883 | 0.89 | 0.889 | 0.895 |
| LRGD0_1 | 0.905 | 0.906 | 0.909 | 0.897 | 0.894 | 0.883 | 1 | 0.974 | 0.975 | 0.968 | 0.956 | 0.964 |
| LRGD0_2 | 0.885 | 0.906 | 0.896 | 0.877 | 0.875 | 0.865 | 0.974 | 1 | 0.956 | 0.953 | 0.936 | 0.948 |
| LRGD0_3 | 0.91 | 0.89 | 0.907 | 0.899 | 0.899 | 0.883 | 0.975 | 0.956 | 1 | 0.966 | 0.959 | 0.965 |
| LRGD1_1 | 0.904 | 0.89 | 0.904 | 0.906 | 0.905 | 0.89 | 0.968 | 0.953 | 0.966 | 1 | 0.98 | 0.984 |
| LRGD1_2 | 0.901 | 0.883 | 0.901 | 0.908 | 0.906 | 0.889 | 0.956 | 0.936 | 0.959 | 0.98 | 1 | 0.981 |
| LRGD1_3 | 0.905 | 0.89 | 0.904 | 0.91 | 0.909 | 0.895 | 0.964 | 0.948 | 0.965 | 0.984 | 0.981 | 1 |
