# Supplementary material for: Transcriptomic Analysis Reveals New Insights into High-Temperature-Dependent Glume-Unclosing in an Elite Rice Male Sterile Line
Source: Front Plant Sci. 2017 Feb 14;8:112. doi: 10.3389/fpls.2017.00112 (PMC5306291; doi:10.3389/fpls.2017.00112)
Supplement: Table S5 — The DEGs enriched in the biological process of carbohydrate metabolism in HRGD1vsHRGD0 and LRGD1vsLRGD0. [file Table5.DOCX]

Table S5 The DEGs enriched in the biological process of carbohydrate metabolism in HRGD1vsHRGD0 and LRGD1vsLRGD0

|  |  | HRGD1vsHRGD0 | LRGD1vsLRGD0 |
| --- | --- | --- | --- |
| up-regulated | common | OS05G0298800,OS01G0946700,OS04G0565400,OS11G0700900,OS11G0701200,OS05G0187100, | |
|  |  | OS01G0946500,OS07G0130100,OS01G0946600,OS04G0486950,OS11G0701400,OS09G0478300, | |
|  |  | OS05G0366600,OS04G0513400,OS09G0491852,OS11G0702100,OS05G0526900 | |
|  | specific | OS08G0244500,OS07G0687900,OS11G0701100, |  |
|  |  | OS11G0701000,OS03G0828300,OS05G0375400, |  |
|  |  | OS05G0247800,OS02G0600400,OS12G0114100, | OS02G0578000,OS07G0543100, |
|  |  | OS06G0328966,OS02G0206700,OS09G0388550, | OS12G0105600,OS01G0865400, |
|  |  | OS01G0944700,OS11G0114600,OS03G0691800, | OS09G0465600,OS10G0545500, |
|  |  | OS03G0841600,OS02G0207100,OS04G0136802, | OS01G0851700,OS04G0164900, |
|  |  | OS01G0713200,OS05G0171900,OS09G0388850, | OS03G0146400,OS06G0713800 |
|  |  | OS11G0153000,OS10G0416100,OS06G0683300, | OS03G0201500,OS09G0478100 |
|  |  | OS03G0184550,OS02G0755900,OS04G0514150, | OS05G0162000,OS01G0201200 |
|  |  | OS02G0654700,OS08G0518900,OS12G0617400, | OS05G0135900,OS10G0189100 |
|  |  | OS02G0725300,OS11G0702200,OS07G0106200, | OS03G0106200,OS09G0491820, |
|  |  | OS06G0288300,OS06G0683100,OS11G0605500, | OS02G0227700,OS09G0271100, |
|  |  | OS04G0506800,OS08G0445700,OS03G0276500, |  |
|  |  | OS07G0539300,OS04G0647800,OS06G0561000, |  |
|  |  | OS12G0231000,OS07G0604000,OS04G0458600, |  |
|  |  | OS05G0363200,OS11G0701800,OS07G0656200, |  |
|  |  | OS03G0255500,OS05G0460000,OS11G0599200, |  |
|  |  | OS06G0676700,OS05G0247100, OS05G0144900, |  |
|  |  | OS01G0170051, OS09G0457400, OS07G0217600, |  |
|  |  | OS03G0277300, OS06G0696600, OS01G0840100 |  |
| Continued |  |  |  |
| down-regulated | common | OS11G0539200,OS02G0752200,OS03G0336400,OS01G0746700,OS02G0575800,OS01G0860450, | |
|  |  | OS01G0799500,OS09G0369400,OS04G0472300,OS07G0529700,OS03G0253200,OS01G0618900, | |
|  |  | OS06G0728800,OS01G0134800,OS06G0554300, OS02G0733300, OS07G0510200, | |
|  | specific |  | OS07G0632000,OS06G0335975,OS03G0722500, |
|  |  |  | OS03G0432277, OS09G0555500,OS01G0726400, |
|  |  |  | OS08G0144100,OS08G0160500,OS07G0480800, |
|  |  |  | OS04G0474700,OS09G0475800,OS03G0710500, |
|  |  | OS12G0614600,OS04G0376400 | OS03G0747900,OS04G0513100,OS06G0531000, |
|  |  | OS12G0151500,OS12G0554800 | OS06G0256900,OS03G0351300,OS09G0394300, |
|  |  | OS04G0631200,OS01G0130400 | OS03G0141200,OS08G0409100,OS02G0589400, |
|  |  | OS05G0587000,OS07G0502900 | OS01G0940800,OS05G0460000,OS04G0678700, |
|  |  | OS09G0538700,OS01G0813800 | OS03G0669300, OS01G0134900, Novel00794, |
|  |  | OS03G0642300,OS05G0332300 | OS07G0598000,OS04G0474500,OS09G0511900, |
|  |  | OS07G0681400,OS04G0231200 | OS02G0790500,OS10G0114400,OS06G0192100, |
|  |  | OS07G0197100 | OS03G0845600,OS02G0773200,OS03G0374600, |
|  |  |  | OS07G0678300,OS03G0386500,OS11G0297800, |
|  |  |  | OS06G0696566, OS11G0673200, OS07G0624600 |
|  |  |  | OS03G0792800, OS03G0711650, OS06G0636600 |
|  |  |  | OS01G0686300, OS10G0492900, OS01G0265100 |
|  |  |  | OS02G0625300, OS04G0394100, OS08G0387400 |
|  |  |  | OS09G0432900,OS09G0433000,OS02G0629200 |
|  |  |  | OS01G0840100, OS03G0277300, OS06G0696600 |

Note: The three genes marked with the red color are common in the up-and down-regulated genes.
